# High *Coxiella burnetii* Seroconversion Rate in Veterinary Students, the Netherlands, 2006–2010

**DOI:** 10.3201/eid2612.200063

**Published:** 2020-12

**Authors:** Marit M.A. de Lange, Wim van der Hoek, Peter M. Schneeberger, Arno Swart, Dick J.J. Heederik, Barbara Schimmer, Inge M. Wouters

**Affiliations:** National Institute for Public Health and the Environment, Bilthoven, the Netherlands (M.M.A. de Lange, W. van der Hoek, A. Swart, B. Schimmer);; Jeroen Bosch Hospital, ‘s-Hertogenbosch, the Netherlands (P.M. Schneeberger);; Utrecht University, Utrecht, the Netherlands (D.J.J. Heederik, I.M. Wouters)

**Keywords:** Q fever, *Coxiella burnetii*, seroepidemiologic studies, seroconversion, veterinarians, students, Netherlands, bacteria, zoonoses

## Abstract

We examined *Coxiella burnetii* seroconversion rates by measuring *C. burnetii* IgG among 2 cohorts of veterinary students. During follow-up of 118 seronegative veterinary students, 23 students seroconverted. Although the clinical importance of the presence of antibodies is unknown, veterinary students should be informed about the potential risks for Q fever.

Q fever is caused by the bacteria *Coxiella burnetii* and can manifest as acute or chronic illness. Veterinarians who care for livestock are prone to *C. burnetii* infection (*1*,*2*). A high seroprevalence among veterinary students has been reported (*3*–*5*). However, the incidence of Q fever and associated risk factors during veterinary training are still unknown. We conducted a longitudinal study at the Faculty of Veterinary Medicine of Utrecht University (FVMUU), Utrecht, the Netherlands, in which we followed incoming, seronegative veterinary students and investigated potential associated factors for seroconversion.

Veterinary students who started in 2006 or 2008 at FVMUU were invited to participate. After obtaining written informed consent, we collected blood samples, and participants completed a baseline questionnaire. From participants who began at FVMUU in 2006 (cohort 2006), <2 additional blood samples and follow-up questionnaires were obtained in 2008 and 2010. Students who started in 2008 (cohort 2008) provided 1 follow-up blood sample and 1 follow-up questionnaire in 2010.

Serum samples were tested for IgG against phase I and II of *C. burnetii*, using an indirect immunofluorescence assay as previously described (*3*). Those samples with IgG phase I or II IgG >1:32 were classified as *C. burnetii* seropositive. Seroconversion was defined as the change observed in a participant who was IgG seronegative at baseline and seropositive in a follow-up sample.

We determined differences in demographics and past animal exposure between seropositive and seronegative participants at baseline. Risk factors for seroconversion were estimated by using univariable logistic regression analyses through generalized estimating equations models (Appendix).

At the beginning of their veterinary training, 447 students were invited to participate in the study. Of those, 131 participated, of whom 13 (10%) were *C. burnetii* IgG seropositive at baseline. Students who were seropositive at baseline were more likely to have lived on a farm and to have had contact with cattle and poultry (Appendix Table 1).

Of the 118 participants seronegative at baseline, 78 started their training in 2006 and 40 in 2008 (Figure). Of those students, 23 seroconverted during the follow-up period of 362 person-years, translating to an incidence of 0.06/person-year. Of the 17 seroconversions in cohort 2006, 11 occurred between baseline and the first follow-up, and 4 occurred between the first and second follow-up (Appendix Table 2). None of the seroconverted participants reported a diagnosis of acute Q fever from a general practitioner or medical specialist, suggesting all cases were mild or asymptomatic. In addition, no participants had serologic indication of a chronic infection. Of the 20 investigated characteristics, “living on a sheep or goat farm,” “having contact with sheep outside [veterinary] training,” and “working with hay, straw, silage grass, or animal feed” outside FVMUU increased the odds of seroconversion (Table).

**Figure:**
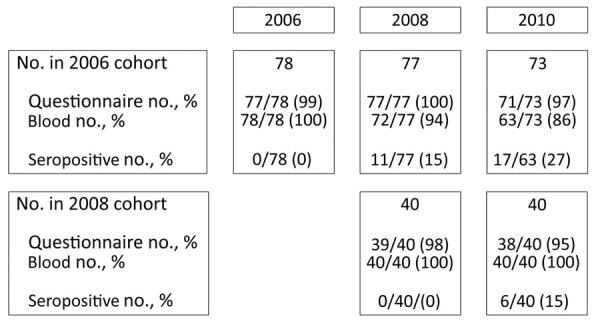
Follow-up timeline illustrating number and percentages of seronegative participants at baseline, per follow-up moment, in study of *Coxiella burnetii* seroconversion rate in veterinary students, the Netherlands, 2006–2010. The 17 seropositive students in 2010 include the 11 students who already seroconverted during 2006–2008 and were censored from risk factor analysis in 2010.

**Table:** Characteristics from follow-up questionnaire in association with *Coxiella burnetii* seroconversion among 118 veterinary students seronegative at baseline, the Netherlands*

Characteristic	Odds ratio (95% CI)	p value
Age group, y		
≤20	Referent	
21	0.9 (0.2–3.5)	0.85
≥22	1.3 (0.4–4.2)	0.69
Sex		
M	Referent	
F	0.7 (0.2–2.3)	0.53
Regular exposure to cigarette smoke		
Yes	1.1 (0.4–2.8)	0.81
No	Referent	
Living on a farm with cattle		
Yes	ND	
No	ND	
Living on a farm with sheep or goats		
Yes	6.2 (1.4–28.1)	0.02
No	Referent	
Living on a farm with pigs		
Yes	ND	
No	ND	
Living on a farm with chickens		
Yes	3.0 (0.3–35.0)	0.39
No	Referent	
Regular contact with cattle outside veterinary training		
Yes	0.3 (0.1–2.7)	0.31
No	Referent	
Regular contact with goats outside veterinary training		
Yes	0.6 (0.1–3.8)	0.56
No	Referent	
Regular contact with horses outside veterinary training		
Yes	0.7 (0.3–1.7)	0.40
No	Referent	
Regular contact with pigs outside veterinary training		
Yes	ND	
No	ND	
Regular contact with chickens outside veterinary training		
Yes	0.5 (0.1–3.8)	0.50
No	Referent	
Regular contact with sheep outside veterinary training		
Yes	4.4 (1.2–16.7)	0.03
No	Referent	
History of performing animal nursing on farm where they lived		
Yes	3.6 (0.9–14.3)	0.07
No	Referent	
History of working with straw or hay on farm where they lived		
Yes	6.4 (1.6–26.1)	<0.01
No	Referent	
History of working with fertilizers on farm where they lived		
Yes	3.2 (0.5–19.6)	0.21
No	Referent	
History of performing plant nursing on farm where they lived		
Yes	3.1 (0.3–33.5)	0.35
No	Referent	
No. years after study start†		
2	Referent	
4	1.0 (0.3–2.9)	0.96
Cohort‡		
2006	Referent	
2008	0.7 (0.3–2.0)	0.56
Chosen specialization during veterinary training		
Individually kept animals	Referent	
Veterinary public health or farm animals	1.6 (0.5–5.0)	0.38

*ND, not determined because of low numbers. †Only adjusted for cohort. ‡Only adjusted for number of years after the study.

We were not able to identify education-related potential risk factors, such as courses taken, for 2 reasons. First, the curriculum changed during our study, so participants from the 2006 and 2008 cohorts took different courses, causing low power in the analysis. Second, within each cohort, little variation occurred in courses taken. Another limitation of this study is our assumption of a constant risk for *C. burnetii* exposure during the study period. Students seem to have been at higher risk for infection in the first 2 study years, although we cannot draw definite conclusions from this small group of students.

Identified risk factors for seroconversion were not education-related. Proximity to (aborting) small ruminants, such as goats and sheep, was a risk factor in an outbreak in the Netherlands (*6*). Veterinary students have a high prevalence of animal contacts outside their education (*7*). In addition, contact with hay, straw, silage grass, or animal feed, is a known risk factor for human Q fever (*8*). A major outbreak of acute Q fever occurred in the Netherlands during 2007–2010 (*9*), and some students might have contracted the infection then, although increased seroprevalence of Q fever in veterinary students before that outbreak has been reported (*3*).

In conclusion, we found a considerable *C. burnetii* seroconversion rate among veterinary students. Although the clinical importance of the presence of antibodies is unknown, students should be advised at the beginning of their education about potential risks and instructed to seek care if they experience symptoms of acute or chronic Q fever infection.

Appendix Additional information about the high *Coxiella burnetii* seroconversion rate in veterinary students, the Netherlands, 2006–2010.
