# Supplementary material for: High Coxiella burnetii Seroconversion Rate in Veterinary Students, the Netherlands, 2006–2010
Source: Emerg Infect Dis. 2020 Dec;26(12):3086–8. doi: 10.3201/eid2612.200063 (PMC7706948; doi:10.3201/eid2612.200063)
Supplement: Appendix — Additional information about the high Coxiella burnetii seroconversion rate in veterinary students, the Netherlands, 2006–2010. [file 20-0063-Techapp-s1.pdf]

Article DOI: https://doi.org/10.3201/eid2612.200063

High *Coxiella burnetii* Seroconversion Rate in Veterinary Students, the Netherlands, 2006–2010

Appendix

Methods

The Study

The Medical Ethical Commission of the University Medical Centre Utrecht approved the study protocol (no. 06/169). Veterinary students who started in 2006 or 2008 (around 225 each year) at the Faculty of Veterinary Medicine of Utrecht University (FVMUU), which is the only Dutch veterinary institution, were invited to participate. After obtaining written informed consent, we collected a blood sample at study start and participants completed a baseline questionnaire. From participants who started at the FVMUU in 2006 (cohort 2006), up to 2 additional blood samples and follow-up questionnaires were obtained in 2008 and 2010. Students who started in 2008 (cohort 2008) provided 1 follow-up blood sample and follow-up questionnaire in 2010.

Questionnaires

Both the baseline and follow-up questionnaires included questions about animal contact, living situation, personal health situation, and smoking habits before and during the study period. The follow-up questionnaires also included questions about focus of study.

Serologic Analysis

Serum samples were tested for IgG antibodies against phase I and II of *C. burnetii*, using an indirect immunofluorescence assay as previously described (*1*). To avoid batch differences, all samples were tested at the end of the study in 1 batch. Those with IgG phase I or II antibodies ≥1:32 were classified as *C. burnetii* seropositive. Seroconversion was defined as a participant who was IgG seronegative at baseline and seropositive in 1 of the follow-up samples.

Participants with an IgG phase I titer of ≥1:1024 had a serologic indication for chronic Q fever infection (*2*).

Statistical Analysis

All data were analyzed with SAS, version 9.4 (SAS Institute Inc., https://www.sas.com/en_us/home.html). First, differences in demographics and past animal exposure characteristics between seropositive and seronegative participants at baseline were determined (Appendix Table 1). To estimate seroconversion rate and possible associated factors for seroconversion during follow-up, data from seronegative participants with at least one follow-up sample were used. The univariable logistic regression analyses were performed with generalized estimating equations models with an exchangeable correlation matrix. These models were used to take into account correlations between the repeated measurements of serostatus within the same subject (*3*). Participants' data were censured for the times after they were tested *C. burnetii* seropositive. The data from the two cohorts were analyzed together because the datasets were too small to analyze them separately. The FVMUU starting year (cohort) and the number of years after the study's beginning were always included as covariates in the model. Investigated characteristics were animal-related exposure outside the study, living situation, smoking habits, study duration, cohort, and chosen area of study; in total, we investigated 20 characteristics. Associations were considered significant at confidence level of $\alpha < 0.05$. All univariable associated characteristics were highly interrelated ($p < 0.05$ in Fisher exact test). Therefore, multivariable logistic analysis with generalized estimating equations was not possible.

References

1. de Rooij MM, Schimmer B, Versteeg B, Schneeberger P, Berends BR, Heederik D, et al. Risk factors of *Coxiella burnetii* (Q fever) seropositivity in veterinary medicine students. PLoS One. 2012;7:e32108. PubMed https://doi.org/10.1371/journal.pone.0032108

2. Wegdam-Blans MC, Kampschreur LM, Delsing CE, Bleeker-Rovers CP, Sprong T, van Kasteren ME, et al.; Dutch Q fever Consensus Group. Chronic Q fever: review of the literature and a proposal of new diagnostic criteria. J Infect. 2012;64:247–59. PubMed https://doi.org/10.1016/j.jinf.2011.12.014

3. Liang K, Zeger SL. Longitudinal data analysis using generalized linear models. Biometrika. 1986;73:13–22. https://doi.org/10.1093/biomet/73.1.13

Appendix Table 1. Baseline questionnaire characteristics of 2 cohorts of veterinary students, the Netherlands*

Characteristic	**Median or no./N (%)** Seronegative participants at baseline, 2006 or 2008, n = 118†	Seropositive participants at baseline, 2006 or 2008, n = 13	**p value**
Age, y	19	18	0.24‡
BMI	21	21	0.87‡
Gender			
M	18/116 (16)	3/13 (23)	0.44
F	98/116 (84)	10/13 (77)	
Smoking status			
Never smoked	109/115 (94)	13/13 (100)	1.00
Past smoker	3/115 (3)	0/13 (0)	
Current smoker	3/115 (3)	0/13 (0)	
Size of place of origin			
Village (<15,000 inhabitants)	44/116 (38)	7/13 (54)	0.45
Town (15,000–80,000 inhabitants)	47/116 (40)	5/13 (38)	
City (>80,000 inhabitants)	25/116 (22)	1/13 (8)	
Ever lived on a farm			
Yes	11/116 (9)	7/13 (54)	<0.01
No	105/116 (91)	6/13 (46)	
Regular contact with cattle before start of FVMUU			
Yes	19/116 (16)	9/13 (69)	<0.01
No	97/116 (84)	4/13 (31)	
Regular contact with goats before start of FVMUU			
Yes	18/116 (16)	5/13 (38)	0.06
No	98/116 (84)	8/13 (62)	
Regular contact with sheep before start of FVMUU			
Yes	20/116 (17)	5/13 (38)	0.13
No	96/116 (83)	8/13 (62)	
Regular contact with poultry before start of FVMUU			
Yes	31/116 (27)	8/13 (62)	0.02
No	85/116 (73)	5/13 (38)	
Regular contact with horses before start of FVMUU			
Yes	65/116 (56)	7/13 (54)	1.00
No	51/116 (44)	6/13 (46)	
Regular contact with pigs before start of FVMUU			
Yes	13/116 (11)	4/13 (31)	0.07
No	103/116 (89)	9/13 (69)	

* BMI, Body Mass Index; FVMUU, Faculty of Veterinary Medicine of Utrecht University; n, Number; N, Total number.
†Two seronegative participants at baseline did not fill out a questionnaire.
‡P value of age and BMI were determined by Fisher Exact test. All other p values shown were determined by Kruskal Wallis test.

Appendix Table 2. Distribution of 2006 cohort, 78 seronegative students at baseline with at least one follow-up sample

Result at 2008 follow-up	Result at 2010 follow-up	No. of students
Positive	Positive	7
Negative	Negative	44
Positive	Negative	3
Negative	Positive	3
Positive	Not tested	1
Negative	Not tested	14
Not tested	Positive	2
Not tested	Negative	4
